# Comparison of the monoamine transporters from human and mouse in their sensitivities to psychostimulant drugs

**DOI:** 10.1186/1471-2210-6-6

**Published:** 2006-03-03

**Authors:** Dawn D Han, Howard H Gu

**Affiliations:** 1 Department of Pharmacology, The Ohio State University College of Medicine, USA; 2 Department of Psychiatry, The Ohio State University College of Medicine, 333 West 10th Avenue, Columbus, Ohio 43210, USA

## Abstract

**Background:**

The plasma membrane neurotransmitter transporters terminate neurotransmissions by the reuptake of the released neurotransmitters. The transporters for the monoamines dopamine, norepinephrine, and serotonin (DAT, NET, and SERT) are targets for several popular psychostimulant drugs of abuse. The potencies of the psychostimulant on the monoamine transporters have been studied by several laboratories. However, there are significant discrepancies in the reported data with differences up to 60-fold. In addition, the drug potencies of the 3 monoamine transporters from mouse have not been compared in the same experiments or along side the human transporters. Further studies and systematic comparisons are needed.

**Results:**

In this study, we compared the potencies of five psychostimulant drugs to inhibit human and mouse DAT, SERT and NET in the same cellular background. The K_I _values of cocaine to inhibit the 3 transporters are within a narrow range of 0.2 to 0.7 μM. In comparison, methylphenidate inhibited DAT and NET at around 0.1 μM, while it inhibited SERT at around 100 μM. The order of amphetamine potencies was NET (K_I _= 0.07–0.1 μM), DAT (K_I _≈ 0.6 μM), and SERT (K_I _between 20 to 40 μM). The results for methamphetamine were similar to those for amphetamine. In contrast, another amphetamine derivative, MDMA (3–4 methylenedioxymethamphetamine), exhibited higher potency at SERT than at DAT. The human and mouse transporters were similar in their sensitivities to each of the tested drugs (K_I _values are within 4-fold).

**Conclusion:**

The current and previous studies support the following conclusions: 1) cocaine blocks all 3 monoamine transporters at similar concentrations; 2) methylphenidate inhibits DAT and NET well but a 1000-fold higher concentration of the drug is required to inhibit SERT; 3) Amphetamine and methamphetamine are most potent at NET, while being 5- to 9-fold less potent at DAT, and 200- to 500-fold less potent at SERT; 4) MDMA has moderately higher apparent affinity for SERT and NET than for DAT. The relative potencies of a drug to inhibit DAT, NET and SERT suggest which neurotransmitter systems are disrupted the most by each of these stimulants and thus the likely primary mechanism of drug action.

## Background

Drug abuse is a serious problem in the United States and around the world that places tremendous social and economical burdens on individuals and on the whole society [1]. Psychostimulants are a group of drugs that stimulate the activity of the central nervous system and produce a series of effects in humans, such as increasing heart rate and respiration, improving alertness, elevating mood and self-confidence, and producing euphoria [2]. Common psychostimulant drugs include: cocaine, methylphenidate (Ritalin), amphetamine, methamphetamine, and MDMA [2]. Some of these psychostimulants are useful medications that have long been used for treating various disorders such as attention deficit hyperactivity disorder (ADHD), narcolepsy, and obesity, while they are also addictive substances that could cause serious adverse effects when abused [2]. Psychostimulant abuse is a major public health problem in the United States. According to the 2003 National Survey on Drug Use and Health [3], cocaine and amphetamine are two of the most abused drugs while methamphetamine abuse has become a growing concern and 12.3 million Americans age twelve and older had tried methamphetamine at least once in their lifetimes. Methylphenidate is commonly prescribed as Ritalin to treat ADHD. As the number of Ritalin prescription increases, drug and law enforcement agencies are seeing an increase in Ritalin drug dealing and the illicit use of Ritalin as a recreational drug [3]. The abuse of MDMA, also known as Ecstasy, has also spread to a wide range of settings and demographic subgroups and more than 10 million people have tried MDMA at least once [3].

Plasma membrane neurotransmitter transporters terminate neurotransmissions by the reuptake and recycling of the released neurotransmitters [4,5]. The transporters for the monoamines dopamine, norepinephrine, and serotonin (DAT, NET, and SERT) belong to a family of Na^+^/Cl^-^-dependent neurotransmitter transporters, which are intrinsic membrane proteins containing 12 putative transmembrane domains [6,7]. Psychostimulants, such as cocaine, methylphenidate, and amphetamine related compounds interrupt the reuptake process by DAT, NET and SERT [8-11]. Consequently, neurotransmissions are prolonged and the extracellular concentrations of these amine transmitters are elevated, resulting in complex neurochemical changes and profound psychiatric effects [12]. In order to understand the effects of these psychostimulant drugs, it is critical to determine which transporter or neurotransmitter systems are most affected at low, medium, or high drug doses. A thorough understanding of the pharmacological profile for each psychostimulant drug would be helpful for the development of treatment protocols for stimulant overdose and dependence.

The potencies of different psychostimulant drugs at the monoamine transporters have been studied and reported by different laboratories [9,10,13-15]. However, there are significant discrepancies among the reported data and the differences are up to 60-fold. For instance, the inhibition constant (K_I_) for amphetamine to inhibit DA uptake in rat synaptosomes was reported to be 0.034 μM in one study [16], while it was reported to be 2.3 μM in cultured cells expressing rat DAT [9]. The MDMA K_I _value to inhibit rat SERT was determined to be 0.24 μM [15] and 2.6 μM [13]. The K_I _values for cocaine to inhibit rat DAT in vitro ranged from 0.33 μM to 2.0 μM [9,17]. These differences are likely due to different experimental procedures employed by each laboratory, the different expression systems or tissue preparation methods used, and different qualities of drugs used.

Therefore, it is important to compare DAT, NET and SERT in their responses to psychostimulants in the same cellular background, using a uniform protocol and drugs. One of our earlier studies compared the cloned DAT, NET and SERT transporters stably expressed in LLC-PK1 cells, but the available transporter cDNAs were from different species [10]. In another study, human clones of the DAT, NET and SERT transporters were stably expressed in HEK293 cells and the uptake inhibition by selected drugs including some psychostimulants were compared [14]. Several other studies examined rat transporters using synaptosomes prepared from rat brains [13,15,18,19]. So far, there is no study focusing on the comparison of psychostimulant potencies among the mouse monoamine transporters and how they compare to the human transporters. With the ever wider use of genetically modified mouse models in recent years, experimental data that compare drug effects between human and mouse monoamine transporters are becoming increasingly important.

In the present study, we compared the potencies of five psychostimulant drugs in inhibiting human and mouse monoamine transporters in the same background and using the same procedure. The drugs examined were cocaine, methylphenidate, amphetamine, methamphetamine, and MDMA. Our results provide new information and confirm most of the published data while differ from some of the previous results. This study, combined with results from other studies, provides very useful information about which neurotransmission pathways are likely to be affected the most by each of the drugs, which gives insight into primary mechanisms of drug actions.

## Results and discussion

**Figure 1:**
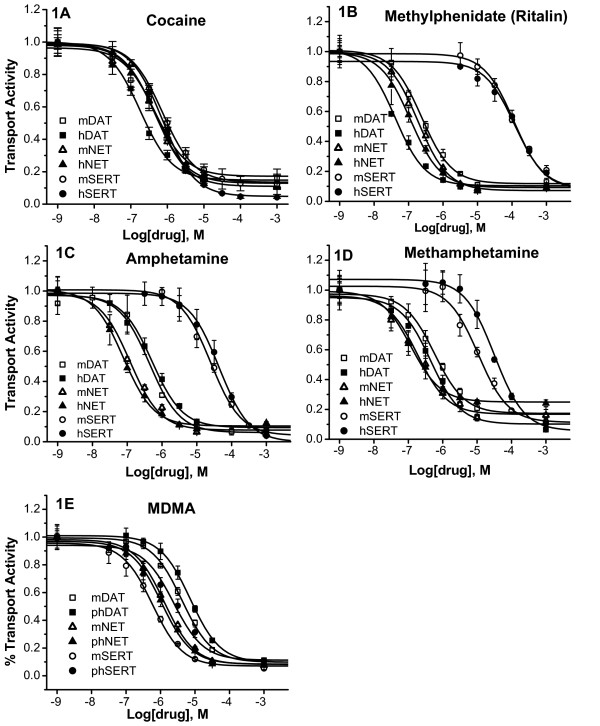
**Drug inhibition profiles of mouse and human monoamine transporters by psychostimulants**. Intestine 407 cells were transfected with human or mouse DAT, SERT, or NET cDNAs. Twenty to 24 hours after transfection, cells were incubated with [^3^H] labeled substrate in PBS/Mg/Ca buffer for 10 min in the absence or presence of increasing concentrations of a psychostimulant drug as indicated. Uptake was terminated by two successive washes with PBS/Mg/Ca. The amounts of [^3^H] labeled substrate accumulated in the cells were determined by scintillation counting. The uptake activities are presented as fractional activities relative to those in the absence of drugs. The experiments were performed in triplicates. Each data point is expressed as mean ± SEM. The five drugs tested are: A) Cocaine; B) Methylphenidate (Ritalin); C) Amphetamine; D) Methamphetamine; and E) MDMA.

Transiently transfected cells are used to determine the K_I _values for drugs inhibiting wild type and mutant monoamine transporters in our laboratory and other laboratories [20-26]. Fluctuations in transporter expression levels have little impact on K_I _measurement except when expression levels are very low resulting in unacceptable signal to noise ratio. In this study, human and mouse DAT, NET, and SERT cDNAs were transiently expressed in cultured Intestine 407 cells. Transport activities by the transfected cells were measured in the presence of increasing concentrations of drugs. The KI values were then determined. Five psychostimulant drugs were studied: cocaine, methylphenidate, amphetamine, methamphetamine, and MDMA. For each of these drugs, the transporters were studied in the same experiments for more precise comparison. Fig. 1 shows representative results for each of the 5 drugs. The results are summarized in Table 1 displaying the average K_I _values from 4–7 experiments. The ratios of highest K_I _values over the lowest were calculated to highlight the differences. The results and conclusions from this study were obtained using Intestine 407 cells, which may not apply to other cell lines or *in vivo *systems.

**Table 1:** Comparison of the K_I _values of 5 psychostimulants to inhibit human and mouse monoamine transporters.

**Drug**	**Cocaine**	**Methylphenidate**	**Amphetamine**	**Methamphetamine**	**MDMA**
Human					
**hDAT**	0.23 ± 0.03	0.06 ± 0.01	0.64 ± 0.14	0.46 ± 0.06	8.29 ± 1.67
**hNET**	0.48 ± 0.05	0.10 ± 0.01	0.07 ± 0.01	0.11 ± 0.01	1.19 ± 0.13
**hSERT**	0.74 ± 0.03	132.43 ± 10.71	38.46 ± 3.84	31.74 ± 2.40	2.41 ± 0.73
**Ratio **^1^	3.2	2207	549	288	7.0
Mouse					
**mDAT**	0.49 ± 0.04 ^2^	0.26 ± 0.03	0.56 ± 0.11	0.47 ± 0.08	4.87 ± 0.65
**mNET**	0.46 ± 0.06 ^2^	0.17 ± 0.03	0.12 ± 0.02	0.19 ± 0.05	1.75 ± 0.51
**mSERT**	0.73 ± 0.12	114.37 ± 7.61	23.82 ± 1.71	9.28 ± 0.86	0.64 ± 0.05
**Ratio **^1^	1.6	672	199	49	7.6

The K_I _values (in μM) were determined as illustrated in Fig. 1. They are expressed as mean ± S.E.M. of four to seven experiments. ^1 ^The ratios of highest K_I _values over the lowest are shown to highlight the differences. ^2 ^The difference between these two K_I _values was not statistically significant (ANOVA *post hoc *Bonferroni test, p > 0.05); all other values were statistically different (ANOVA comparing the three transporters within the same species and for the same drug).

### Cocaine

Fig. 1A shows that cocaine inhibited DAT, SERT, and NET from human or mouse within a narrow range of concentrations. The K_I _values were from 0.2 to 0.7 μM. Among the human monoamine transporters, cocaine was a slightly more potent inhibitor of hDAT (K_I _= 0.23 μm) than hSERT (K_I _= 0.74 μm) with hNET in the middle (K_I _= 0.48 μm). The cocaine potencies that inhibit mouse transporters were very similar to those for the human transporters except that the K_I _for mDAT (0.49 μm) was about twice the value for hDAT. Compared to earlier studies, our K_I _value (0.23 μM) for hDAT was similar to the K_I _value (0.278 μM) reported by Eshleman, et al. for hDAT stably expressed in HEK293 cells [14] but 4 times the value (K_I _= 0.058 μM) reported by Giros, et al. for hDAT expressed in mouse fibroblast Ltk-cells [27]. For both human and mouse NET and SERT, our data indicated that NET is slightly more sensitive to cocaine than SERT, while two previous studies using rat synaptosomes or human transporters expressed in cultured cells [14,15] suggest that SERT is slightly more sensitive to cocaine than NET. Despite the small differences, all studies show that cocaine inhibits DAT, NET and SERT within a narrow concentration range, suggesting that modulation of all three neurotransmitter systems are likely to contribute to the biochemical and behavioural effects of cocaine.

### Methylphenidate (Ritalin)

As shown in Fig. 1B and Table 1, methylphenidate was a very potent inhibitor of hDAT and hNET (K_I _= 0.06 μM and 0.10 μM), while it was not a potent inhibitor of hSERT (K_I _= 132 μM). Therefore, hSERT is over 2000 fold less sensitive to methylphenidate than hDAT. Comparison between the two species revealed that methylphenidate was 4-fold more potent to inhibit hDAT than mDAT, while it had similar effects on NET or SERT from the two species. Our data show trends similar to those previously reported for human transporters expressed in HEK cells [14] and for rat transporters studied with rat brain synaptosomes [13]. For instance, the methylphenidate K_I_values determined by Eshleman et al are 0.19 μM for hDAT, 0.038 μM for hNET and 55 μM for hSERT [14]. The common conclusion from all these studies is that SERT is much less sensitive to methylphenidate than DAT and NET.

Methylphenidate is marketed as Ritalin and is prescribed to treat ADHD particularly for children. This prescription drug is also addictive and has been abused. It has been reported that cocaine and methylphenidate accumulate in the same regions in the human brain and have similar effectiveness in blocking DAT in vitro and in vivo [28]. However, methylphenidate abuse by humans is much less frequent than that for cocaine [29]. The major difference between methylphenidate and cocaine is that SERT is not significantly inhibited by methylphenidate with doses that completely block DAT and NET while cocaine blocks all three transporters equally well. This difference likely contributes to the different effects produced by the two drugs. Another likely contributing factor is the different pharmacokinetics for the two drugs with cocaine reaching its site of action much more rapidly than methylphenidate.

### Amphetamine and methamphetamine

Among human transporters, amphetamine was most potent at inhibiting hNET (K_I _= 0.07 μM). Compared to the amount of amphetamine required to inhibit hNET, 9-fold and over 500-fold more amphetamine was required to inhibit hDAT (K_I _= 0.64 μM) and hSERT (K_I _= 38 μM) respectively. The potencies of methamphetamine (Fig. 1D) to inhibit human and mouse monoamine transporters are similar to those of amphetamine (Fig. 1C). We compared the human and mouse monoamine transporters and found that the transporters from the two spices responded to amphetamine and methamphetamine in a similar fashion (Fig. 1C, 1D, and Table 1). Our results are not the same as those from previous studies but show a similar trend. In one study with rat synaptosomes, the amphetamine K_I _values are 0.034 μM for rDAT, 0.039 μM for rNET, and 3.8 μM for rSERT [15]. In another study using cultured cells expressing human transporters, the methamphetamine K_I _values are 0.082 μM for hDAT, 0.0013 μM for rNET, and 20.7 μM for hSERT. Therefore, amphetamine and methamphetamine are at least 100-fold less potent at inhibiting SERT than DAT or NET.

Amphetamine is a synthetic drug. While prescribed for treating ADHD, amphetamine has also been frequently diverted from prescription to recreational use. Methamphetamine is an amphetamine analogue which also has some limited therapeutic uses, primarily in the treatment of ADHD and obesity. In recent years, the abuse of methamphetamine becomes an extremely serious and growing problem. Amphetamine and its analogues are also substrates of the monoamine transporters and the vesicular monoamine transporter. They compete with and exchange with monoamines at the plasma membrane transporters and also at the vesicular monoamine transporter, disrupting the reuptake process and causing the release of monoamines [7,30]. Amphetamine induced monoamine release is usually studied using animal brain preparations which contain both the plasma membrane and vesicular monoamine transporters. Cultured cells expressing only plasma membrane transporters are usually used to measure the K_I _values of uptake inhibition which reflect the apparent affinities of the drugs to each transporter. The K_I _value includes the effect of amphetamine induced substrate release and it is not exactly the same as the dissociation constant K_D_, a true measurement of drug affinity. In this study, we focused on comparing K_I _values of the transporters and did not study drug induced releases. The results show that amphetamine and methamphetamine are most potent in inhibiting NET and much less potent in inhibiting SERT in both human and mouse.

### MDMA

As its name indicated, 3,4-methylenedioxymethamphetamine is a compound with a methylenedioxy group added to methamphetamine. The chemical modification substantially increases MDMA's potency to inhibit SERT while reducing its potencies to inhibit DAT and NET compared to methamphetamine. This brings the K_I _values for all three monoamine transporters to a close range (7-fold). In mouse, the order of potencies for MDMA was SERT (K_I _= 0.64 μM), NET (K_I _= 1.75 μM), and DAT (K_I _= 4.87 μM). For human transporters, the order was NET (K_I _= 1.19 μM), SERT (K_I _= 2.41 μM), and DAT (K_I _= 8.29 μM). In previous studies, Rothman, et al. has reported that among the rat transporters, MDMA is most potent at inhibiting rSERT (K_I _= 0.238 μM), followed by rNET (K_I _= 0.462 μM) and rDAT (K_I _= 1.572 μM) [15]. However, another study has reported that MDMA is more potent at inhibiting rDAT (K_I _= 1.53 μM) than rSERT (K_I _= 2.6 μM) [13]. Our data for the mouse and human transporters are similar to the results by Rothman et al for rat transporters [15]. Our results indicate that MDMA is more potent in inhibiting SERT than DAT, which is in contrary to the other amphetamine derivatives.

## Conclusion

There are significant discrepancies in previous studies on the potencies of psychostimulant drugs at monoamine transporters, likely due to differences in experimental setups, expression systems, tissue preparations, and/or drug qualities. In this study, we compared the potencies of five commonly abused psychostimulants at the human and mouse DAT, SERT and NET in the same cellular background. Cocaine blocked the 3 monoamine transporters at similar concentrations (K_I _= 0.2–0.7 μM). In comparison, methylphenidate inhibited DAT and NET around 0.1 μM, while inhibited SERT at 1000 fold higher concentration (around 100 μM). Amphetamine and methamphetamine were most potent for NET (K_I _around 0.1 μM), less potent for DAT (K_I _around 0.5 μM), and much less potent for SERT (K_I _between10 to 40 μM). In contrast, MDMA, another amphetamine derivative, exhibited higher potency at SERT than at DAT. The human and mouse transporters were similar in their sensitivities to each of the tested drugs (K_I _values within 4 folds). The relative potencies of a drug in inhibiting DAT, NET and SERT suggest the neurotransmitter systems that are disrupted the most and thus the primary mechanism of drug action.

## Methods

### Materials

Cocaine, D-amphetamine, and methylphenidate were kindly provided by National Institute on Drug Abuse through its Drug Supply Program. D-methamphetamine and MDMA were purchased from Sigma (St Louis, MO). [^3^H]-labelled dopamine (23.5 Ci/mmole) and [^3^H]-labelled serotonin (27.1 Ci/mmole) were purchased from PerkinElmer Life and Analytical Sciences (Boston, MA). The human dopamine, norepinephrine, and serotonin transporter cDNAs used in the experiments were described previously [10,31,32]. The cloning of mouse DAT cDNA was reported in an earlier publication [33]. The mouse NET and SERT cDNAs were amplified with nested PCR using mouse brain cDNAs as the template. The forward primers for mNET are: mNETf1 (CAGCCGCACCCATGCTTCT) and mNETf2 (AAAAGGTACCACCATGCTTCTGGCGCGAAT); the reverse primers are mNETr1 (TCCTCCACATTGCCAGGTTCAGA) and mNETr2 (AAAATCTAGAGGTTCAGATGGCCAGCCAGTG). The forward primers for mSERT are mSTf1 (AGCTAGTCAGGGTCCTTGGCAGATG) and mSTf2 (ATATCCATGGAGACCACACCTTTGAATTCTC), and reverse primers are mSTr1 (TGGGGCTTTTCAGAGATGAGGAGTC) and mSTr2 (TAATCTCGAGCCATGTCCTCTCCCTCAGTGTGTTAC). Restriction enzyme sites were incorporated in the primers for insertion into plasmid vectors. Oligonucleotide primers were synthesized by commercial DNA synthesis services. The correct sequences of mNET and mSERT were confirmed by sequence determination.

### Transient expression of the transporter cDNAs

The transporter cDNAs were subcloned into the bluescript vector SKII+ (Strategene, La Holla, CA) which has a T7 promoter. The cDNAs were transiently expressed in monkey Intestine 407 cells (CCL-6, American Type Culture Collection, Rockville, MD) and characterized as described [21,33]. Briefly, cells were plated in 96-well plates, transfected with plasmid DNA using Lipofectin (Invitrogen Life Technologies, Carlsbad, CA) according to manufacturer's instruction, and infected with a recombinant vaccinia virus VTF-7 which carries the T7 polymerase gene [34].

### Transport measurement and drug inhibition

After 20- to 24-hour incubation, the transfected cells were washed once with PBS/Ca/Mg (phosphate buffered saline solution supplemented with 1 mM MgCl_2_, 0.1 mM CaCl_2_), and then incubated for 10 minutes at 20°C in the same buffer containing 50 nM [^3^H]-labelled substrate, 50 μM L-ascorbic acid (to protect the substrates from being oxidized), and different concentrations of the tested drugs (as described in Results or in each figure legend). At the end of the incubation, cells were washed 2 times with PBS/Ca/Mg, and then dissolved in 0.1 M NaOH. The amount of accumulated [^3^H]-labelled substrate in the cells were determined by counting in scintillation fluid (MicroScint-20, PerkinElmer Life Sciences, Boston, MA) using a Packard TopCount, a microplate scintillation and luminescence counter. All experiments were performed in triplicates.

### Data analysis

The IC_50 _values were determined by nonlinear regression of experimental data for each experiment according to a hyperbolic model using the computer program Origin (MicroCal Software, Northampton, MA). The K_I _values were then calculated from the IC_50 _values using the equation K_I _= IC_50_/(1 + [S]/K_M_). Data are presented as arithmetic mean ± SEM of four to seven independent experiments. ANOVA was used to determine statistical significance.

## Authors' contributions

DDH carried out most of the experiments and data analyses, and drafted the manuscript. HHG conceived of the study, and participated in its design and coordination and helped to draft the manuscript. All authors read and approved the final manuscript.
